# Ets and GATA Transcription Factors Play a Critical Role in PMA-Mediated Repression of the *ckβ* Promoter via the Protein Kinase C Signaling Pathway

**DOI:** 10.1371/journal.pone.0113485

**Published:** 2014-12-09

**Authors:** Chee Sian Kuan, Yoke Hiang Yee, Wei Cun See Too, Ling Ling Few

**Affiliations:** School of Health Sciences, Health Campus, Universiti Sains Malaysia, 16150 Kubang Kerian, Kelantan, Malaysia; Hokkaido University, Japan

## Abstract

**Background:**

Choline kinase is the most upstream enzyme in the CDP-choline pathway. It catalyzes the phosphorylation of choline to phosphorylcholine in the presence of ATP and Mg^2+^ during the biosynthesis of phosphatidylcholine, the major phospholipid in eukaryotic cell membranes. In humans, choline kinase (CK) is encoded by two separate genes, *ckα* and *ckβ*, which produce three isoforms, CKα1, CKα2, and CKβ. Previous studies have associated *ckβ* with muscle development; however, the molecular mechanism underlying the transcriptional regulation of *ckβ* has never been elucidated.

**Methodology/Principal Findings:**

In this report, the distal promoter region of the *ckβ* gene was characterized. Mutational analysis of the promoter sequence and electrophoretic mobility shift assays (EMSA) showed that Ets and GATA transcription factors were essential for the repression of *ckβ* promoter activity. Supershift and chromatin immunoprecipitation (ChIP) assays further identified that GATA3 but not GATA2 was bound to the GATA site of *ckβ* promoter. In addition, phorbol-12-myristate-13-acetate (PMA) decreased *ckβ* promoter activity through Ets and GATA elements. PMA also decreased the *ckβ* mRNA and protein levels about 12 hours after the promoter activity was down-regulated. EMSA further revealed that PMA treatment increased the binding of both Ets and GATA transcription factors to their respective DNA elements. The PMA-mediated repressive effect was abolished by chronic PMA treatment and by treatment with the PKC inhibitor PKC412, but not the PKC inhibitor Go 6983, suggesting PKCε or PKCη as the PKC isozyme involved in the PMA-mediated repression of *ckβ* promoter. Further confirmation by using PKC isozyme specific inhibitors identified PKCε as the isozyme that mediated the PMA repression of *ckβ* promoter.

**Conclusion/Significance:**

These results demonstrate the participation of the PKC signaling pathway in the regulation of *ckβ* gene transcription by Ets and GATA transcription factors.

## Introduction

Choline kinase (CK) (EC 2.7.1.32) uses Mg^2+^ as a cofactor to catalyze the ATP-dependent phosphorylation of choline, yielding phosphocholine (PCho) and ADP. CK commits choline to the Kennedy pathway for the *de novo* biosynthesis of phosphatidylcholine (PC) [1]. PC, also known as lecithin, is the most abundant membrane phospholipid (40–60%) in eukaryotic cells [1]. Apart from being a component of the membrane architecture, PC has also been linked to mitogenic signaling by serving as a substrate for the production of mitogenic signaling molecules, such as diacylglycerol (DAG) and phosphatidic acid (PA), which are generated from the hydrolysis of PC by phospholipase C and phospholipase D, respectively [2], [3].

CK was first discovered as a cytosolic enzyme in brewer's yeast [4]. It was later found in other organisms, ranging from yeast to mammals [5], [6]. In humans, CKs are encoded by two separate genes, *ckα* and *ckβ*. Based on their high sequence homology, *ckα* and *ckβ* might have appeared after genetic duplication from a common ancestor [7]. The expression of these two genes results in three different proteins, CKα1 (439 amino acids; NCBI accession number NP_997634), CKα2 (457 amino acids; NCBI accession number NP_001268), and CKβ (395 amino acids; NCBI accession number NP_005189) [8]. CKα1 and CKα2 are splice variants derived from the primary *ckα* mRNA, while CKβ is a separate product of the *ckβ* gene [8].

Apart from its traditional role in phospholipid biosynthesis, CKβ is also involved in muscle development [9],[10]. Deletion of the murine *ckβ* gene causes rostrocaudal muscular dystrophy (RMD), which is characterized by neonatal forelimb bone deformity and progressive muscle wasting [9], [10]. RMD mice display a high abundance of abnormally enlarged mitochondria at the periphery of cells [11]. In *ckβ* knockout mice, the decreased levels of PC, along with the decreased ATP synthesis caused by deficiencies in complex III of the electron transport chain, result in mitochondrial dysfunction and subsequent significant mitochondrial loss through mitophagy [11]. In humans, genetic mutation of the *ckβ* gene and consequent altered PC biosynthesis is the primary cause of congenital muscular dystrophy (CMD). CMD patients have reduced levels of PC and CK activity due to a genetic defect in the *ckβ* gene [12].

Despite the importance of *ckβ* in PC synthesis, mitochondrial function, and muscular dystrophy, literature describing the transcriptional regulation of the *ckβ* gene is still lacking. Important *cis*-acting regulatory elements of the *ckβ* gene promoter have yet to be identified. The molecular mechanisms that regulate *ckβ* expression can be elucidated by characterization of the *ckβ* promoter. Previous studies in various mammalian cells showed that phorbol esters stimulate the incorporation of choline into PC [13], [14]. Phorbol 12-myristate 13-acetate (PMA) is a direct activator of protein kinase C (PKC), and stimulates both the cellular uptake of radiolabeled choline and its incorporation into PC [15], [16]. Previously, we isolated a 2000 bp human *ckβ* promoter that was repressed by PMA treatment [17]. In this report, we localized the repressive effect of PMA to the −2000/−1886 region upstream of the ATG translation start site, which is bound by Ets and GATA transcription factors. We also demonstrate that PMA exerts its effect on the *ckβ* promoter through a PKC-dependent pathway.

## Materials and Methods

### *In silico* analysis of the *ckβ* promoter region

The 2000 bp upstream region of the *ckβ* gene (transcript NM_005198) was analyzed using MatInspector 8.0 [18] and TFSEARCH [19] to identify putative transcription factor binding sites. CpG islands within the *ckβ* promoter (the 2000 bp region upstream of the *ckβ* gene) were identified using CpGplot [20] and CpGIS [21]. CpGplot defines a CpG island as a DNA region with an observed/expected ratio >0.60, a length >200 bp, and GC content >50% [20], [22]. CpGIS defines a CpG island as a sequence having an observed/expected ratio >0.65, a length >500 bp, and GC content >55% [21].

### Cell culture

The human liver carcinoma cell line HepG2 (ATCC No. HB-8065), the human colorectal carcinoma cell line, HCT116 (ATCC no. CCL-247) and the human breast adenocarcinoma cell line MCF-7 (ATCC No. HTB-22) were cultured in high-glucose Dulbecco's modified Eagle's medium (DMEM) supplemented with 10% (v/v) heat-inactivated fetal bovine serum (FBS), 100 U/mL penicillin, and 100 µg/mL streptomycin. Cells were maintained at 37°C in a humidified atmosphere of 5% (v/v) CO_2_.

### Plasmid constructs

The *ckβ* promoter fragment −2000/−1 was amplified by PCR from human genomic DNA (Roche, Germany) using a forward primer [*ckβ*-2000-5′] and reverse primer (*ckβ*-3′) that incorporate *Xho*I and *Hind*III restriction sites, respectively. The PCR product was cloned in-frame at the 5′ *Xho*I*/Hind*III site of the promoterless firefly luciferase reporter vector pGL4.10[*luc2*] (Promega, USA), yielding pGL4.10-*ckβ*(−2000/−1). The 5′-end deletion constructs of pGL4.10-*ckβ*(−1886/−1), pGL4.10-*ckβ*(−1770/−1), pGL4.10-*ckβ*(−1651/−1), pGL4.10-*ckβ*(−1477/−1), pGL4.10-*ckβ*(−914/−1), and pGL4.10-*ckβ*(−519/−1) were generated by PCR using pGL4.10-*ckβ*(−2000/−1) as the template and subcloned into the *Xho*I*/Hind*III site of the pGL4.10[*luc2*] vector. The primers used are listed in Table 1. All deletion mutant constructs generated by PCR were verified by DNA sequencing.

**Table 1:** Primers used for cloning of promoter-luciferase constructs, PCR-based site-directed mutagenesis, real-time PCR and ChIP.

Name	Sequence 5′ to 3′	Orientation
**Promoter-luciferase constructs**		
*ckβ*-2000-5′	CCGCTCGAGATGATGCTTCAGGGCTCC	Forward
*ckβ*-1886-5′	CCGCTCGAGGCTGTTCAGACAGTCTTGCTG	Forward
*ckβ*-1770-5′	CCGCTCGAGGCTGGAACCACAGGCACCTG	Forward
*ckβ*-1651-5′	CCGCTCGAGTCGGCTTCCCAAAGTGCAGG	Forward
*ckβ*-1477-5′	CCGCTCGAGCGTGTAGCTGGGATTACAGGC	Forward
*ckβ*-914-5′	CCGCTCGAGCTCAATGGGAGGTGCGCAGG	Forward
*ckβ*-519-5′	CCGCTCGAGTGGACGGCTCTTCCTTGTCGG	Forward
*ckβ-3*′	CCCAAGCTTGGCGCGGGCTCGACCGGG	Reverse
**Site-directed mutagenesis**		
mut-Ets	CCGCTCGAGATGATGCTTCAGGGCTCCTGGAAACAGTGTCAGCTC*gt*A*cg*CTGTATCAGCCCTTC	Forward
mut-GATA	CCGCTCGAGATGATGCTTCAGGGCTCCTGGAAACAGTGTCAGCTCAGATCCTGT*gc*CA*tt*CCTTCAAAGACCTAG	Forward
mut-Ets/GATA	CCGCTCGAGATGATGCTTCAGGGCTCCTGGAAACAGTGTCAGCTC*gt*A*cg*CTGT*gc*CA*tt*CCTTCAAAGACCTAG	Forward
*ckβ-*3′	CCCAAGCTTGGCGCGGGCTCGACCGGG	Reverse
**Real-time PCR**		
*ckβ-*Fwd	ATGTTCGCCATACTTGCGGA	Forward
*ckβ-*Rvr	AATTGCGCCATCTTCGTGG	Reverse
UBC-Fwd	TTCTTGATCCCCAATGCTTC	Forward
UBC-Rvr	AGTTAAGGGCCAGACCCAGT	Reverse
YWHAZ-Fwd	CTGATCAGCAGAGGTTGATCTT	Forward
YWHAZ-Rvr	GTCTTGCCAGTGAGTGTCTT	Reverse
**ChIP**		
*ckβ*-2000-5′	CCGCTCGAGATGATGCTTCAGGGCTCC	Forward
*ckβ*-Distal-3′	CCAGCCTGAGCAACACAGC	Reverse

The mutations introduced into the binding sites are italicized and in lowercase.

### Site-directed mutagenesis

Mutation of the Ets and GATA consensus binding sites was carried out by one-step PCR mutagenesis using Platinum *Pfx* DNA polymerase (Invitrogen, USA). Primers used to introduce the mutations are shown in Table 1. The pGL4.10-mut(Ets), pGL4.10-mut(GATA), and pGL4.10-mut(Ets/GATA) mutant constructs were verified by DNA sequencing.

### Transient transfection and luciferase assay

Transfection was performed using Lipofectamine 2000 (Invitrogen/Life Technologies) according to the manufacturer's instructions. Briefly, MCF-7 cells were plated in 100 µL of medium/well on a 96-well plate at a density of 1.5×10^4^ cells/well for 24 hr. The cells in each well were transfected with 200 ng of pGL4.10[*luc2*] or the various promoter-luciferase constructs and 2.5 ng of the pGL4.73[*hRluc*/SV40] (Promega) vector, as the control for transfection efficiency. Forty-eight hours after transfection, cells were harvested and assayed using the Dual-Glo Luciferase Reporter Assay System (Promega). The luminescent signals were measured on a GloMax 20/20 luminometer (Promega, USA). The luciferase assays were performed in triplicate in three independent experiments.

### PMA, PKC412, Go 6983 and PKC isozyme specific inhibitors treatments

MCF-7 cells were seeded in 96-well plates overnight and then transfected with pGL4.10-*ckβ*(−2000/−1) and pGL4.73[*hRluc*/SV40]. Six hours after transfection, the culture medium was replaced with 1% (v/v) serum-free DMEM for 20 hr before stimulation. The cells were treated with 10, 20, or 30 ng/mL PMA (EMD Chemicals, USA) for 6 hr, except for the time-course study, in which cells were treated with 20 ng/mL PMA for 6 to 48 hr. For controls, DMSO was added to the cells instead of PMA. For the identification of PKC isozyme involved in the PMA-mediated repression of the *ckβ* promoter, MCF-7 cells were individually or co-treated with PMA (20 ng/mL) and a PKC inhibitor [1 mM PKC412 (Tocris Bioscience, USA), 0.1 mM Go 6983 (Tocris Bioscience, USA), 10 µM PKCε inhibitor peptide (Santa Cruz, USA) or 10 µM PKCη pseudo-substrate inhibitor (Santa Cruz, USA)] for 6 hr. After the treatments, luciferase activities were measured using the Dual-Glo luciferase assay. To determine whether the PMA response element is localized to the Ets and GATA binding sites, pGL4.10-mut(Ets), pGL4.10-mut(GATA), and pGL4.10-mut(Ets/GATA) mutant constructs were individually transfected into MCF-7 cells, followed by incubation with 20 ng/mL PMA for 6 hr before luciferase activity was determined.

### Quantitative real-time PCR of *ckβ*

Total RNA from PMA treated (20 ng/mL for 6 hr) or untreated (DMSO) cells were extracted using the RNeasy Mini Kit (Qiagen, USA) according to the manufacturer's instructions. One microgram of RNA was reverse transcribed by using the RevertAid H Minus First Strand cDNA synthesis kit (MBI Fermentas, USA). The mRNA level of *ckβ* was measured by quantitative real-time PCR (qPCR) performed with an ABI PRISM 7000 Sequence Detection System (Applied Biosystems, USA). The intron-spanning primers used to amplify *ckβ* and the primers for the reference genes (UBC and YWHAZ) are listed in Table 1. The PCR efficiencies for all the primers were higher than 90%. Each reaction was performed in a 25 µL volume containing 12.5 µL Power SYBR Green I Master Mix (Applied Biosystems, USA), 300 nM of each primer and 1 µL of 1∶2 diluted cDNA as template. The cycling program was the default settings of the ABI PRISM 7000 SDS software as follows: 2 min at 50°C, 10 min at 95°C, followed by 40 cycles of 10 sec at 95°C and 1 min at 60°C. Melting curve analysis was carried out immediately after the amplification with temperatures ranging from 60 to 95°C in 0.1°C increments to verify the PCR specificity. The obtained *C*t value was normalized with the geometric mean of UBC and YWHAZ reference genes [23]. The expressions of *ckβ* gene in PMA treated cells relative to control (DMSO treated) cells were calculated by the ΔΔ*C*t method whereby ΔΔ*C*t =  (*C*t_target gene, test sample_ – *C*t_endogenous control, test sample_) – (*C*t_target gene, calibrator sample_ – *C*t_endogenous control, calibrator sample_) and fold change  = 2^−ΔΔ*C*t^ [24].

### Western detection of CKβ

Thirty micrograms of cell lysate were separated on 10% SDS-PAGE and electroblotted at 13 volts for 2 hr onto nitrocellulose membrane. After the transfer step, the nitrocellulose membrane was immersed in blocking buffer (10 mM Tris-HCl, pH 7.5 containing 5% (w/v) skim milk, 150 mM NaCl and 0.1% (v/v) Tween 20) for 1 hr with gentle agitation at room temperature. Next, the membrane was incubated with 1∶1000 diluted CKβ antibody [2] in the blocking buffer at 4°C for overnight. The membrane was washed 3 times with Tris-buffered saline for 1 hr before incubation with 1∶5000 diluted HRP-conjugated rabbit IgG secondary antibody for 1 hr at room temperature. Subsequently, the membrane was washed 3 times for 30 min with Tris-buffered saline and the signal was detected with SuperSignal West Pico chemiluminescent substrate (Thermo Scientific, USA) and Fusion FX luminescence detector system (Vilber Lourmat, France). For loading control, the same membrane was stripped with stripping buffer (200 mM glycine, pH 2.5 containing 0.1% (w/v) SDS and 1% (v/v) Tween 20) and re-probed with 1∶1000 diluted β-actin antibody (Abcam, USA). Signal intensities on the blots were analyzed by using ImageJ version 1.49b software (downloaded from http://imagej.nih.gov/ij/). The integrated densities of bands were measured in triplicate and the average values were then normalized to the corresponding integrated densities of β-actin signals.

### Electrophoretic mobility shift assays (EMSAs)

Nuclear extract from MCF-7 cells was prepared using the NE-PER nuclear and cytoplasmic extraction kit (Pierce, USA) according to the manufacturer's protocol. All DNA probes used in EMSAs were synthesized by 1st Base (Malaysia) and labeled with biotin using a biotin 3′ end DNA labeling kit (Pierce). Complementary DNA probes were annealed with each other at a 1∶1 molar ratio by heating at 95°C for 5 min, followed by a gradual decrease to room temperature using a thermocycler set to decrease 1°C per cycle. EMSAs were performed using the LightShift chemiluminescent EMSA kit (Pierce). The binding reaction mixture contained 200 fmol biotin-labeled probe, 10 µg of nuclear extract, 1× binding buffer, 2.5% glycerol, 5 mM MgCl_2_, 1 µg poly(dI–dC), 2 µg BSA, and 0.05% NP-40. For the competition assay, 100-fold unlabeled double-stranded DNA was added into the reaction mixture. DNA probes used in EMSAs are listed in Table 2. For the supershift assay, 5 µg of GATA2 (Abcam, United Kingdom) or GATA3 (Millipore, USA) antibody was added into the reaction mixture and incubated on ice for 30 min prior the addition of the biotinylated probe. DNA-protein complexes were resolved by electrophoresis on a 6% (w/v) non-denaturing polyacrylamide gel in 0.5× Tris-borate-EDTA (TBE) at 4°C. The gel was run until the bromophenol blue dye has reached 3/4 of the length of the gel for EMSA or until the dye has completely migrated out of the gel for supershift assay (to increase the possibility of detecting slower-migrating complexes). The biotin-labeled DNA was electro-blotted onto a Biodyne B nylon membrane (Pierce) and the membrane was cross-linked by a UV transilluminator (Spectronics, USA) at 312 nm for 15 min. The signal was then developed with a chemiluminescent nucleic acid detection module (Pierce). The images were developed by exposing the signal to X-ray film for 2–5 min or detected with Fusion FX luminescence detector system (Vilber Lourmat, France).

**Table 2:** DNA probes used in EMSA. The Ets and GATA core sequences are underlined.

Name	Sequence 5′ to 3′	References
Biotin-*ckβ*-Ets/GATA	TGTCAGCTCAGATCCTGTATCAGCCCTTCAAAG	This study
Biotin-*ckβ*-mut-GATA	TGTCAGCTCAGATCCTGTGCCATTCCTTCAAAG	This study
Biotin-*ckβ*-mut-Ets	TGTCAGCTCGTACGCTGTATCAGCCCTTCAAAG	This study
Biotin-*ckβ*-mut-Ets/GATA	TGTCAGCTCGTACGCTGTGCCATTCCTTCAAAG	This study
Ets consensus probe	GGCCAAGCCGGAAGTGAGTGC	[25]
Ets/GATA consensus probe	GATCTCCGGCAACTGATAAGGATTCCCTG	[26],[27],[28]

### Chromatin immunoprecipitation (ChIP)

Chromatin immunoprecipitation (ChIP) assay was performed using Pierce Agarose ChIP Kit (Thermo Fisher Scientific, USA) according to the manufacturer's instructions. MCF-7 cells were treated with 1% (v/v) formaldehyde (Sigma-Aldrich, USA) for 10 min to induce protein-DNA cross-linking. Glycine solution (1×) was added to the mixture, incubated for 5 min and the cells were washed with PBS twice. The cells were harvested by scraping, then pelleted and resuspended in 400 µL of lysis buffer. Samples were centrifuged and the nuclear pellet was mixed with 400 µL of micrococcal nuclease (MNase) solution at 37°C for 15 min. The reaction was stopped by adding 40 µL of MNase Stop Solution and the samples were centrifuged to prepare the supernatants (digested chromatin) for immunoprecipitation. The DNA-protein complexes were immunoprecipitated at 4°C for overnight with 10 µg of GATA2 or GATA3 antibody. Immunoprecipitation with 2 µL of pre-immune normal rabbit IgG was used as control. DNA recovered from the immunoprecipitated samples was PCR amplified by using *ckβ*-2000-5′ and *ckβ*-Distal-3′ primers (Table 1) followed by agarose gel electrophoresis. The positive PCR product was verified by sequencing.

### Statistical analysis

Data were analyzed using Student's *t-*test or one-way ANOVA, followed by the Tukey Honestly Significant Differences (HSD) *post-hoc* test. The analysis was performed using PASW Statistics 18, Release Version 18.0.0 (SPSS, Inc., 2001, USA). Data are presented as the mean ±SEM of at least three independent experiments.

## Results

### The human *ckβ* promoter is a TATA-less, CpG island-containing promoter

**Figure 1:**
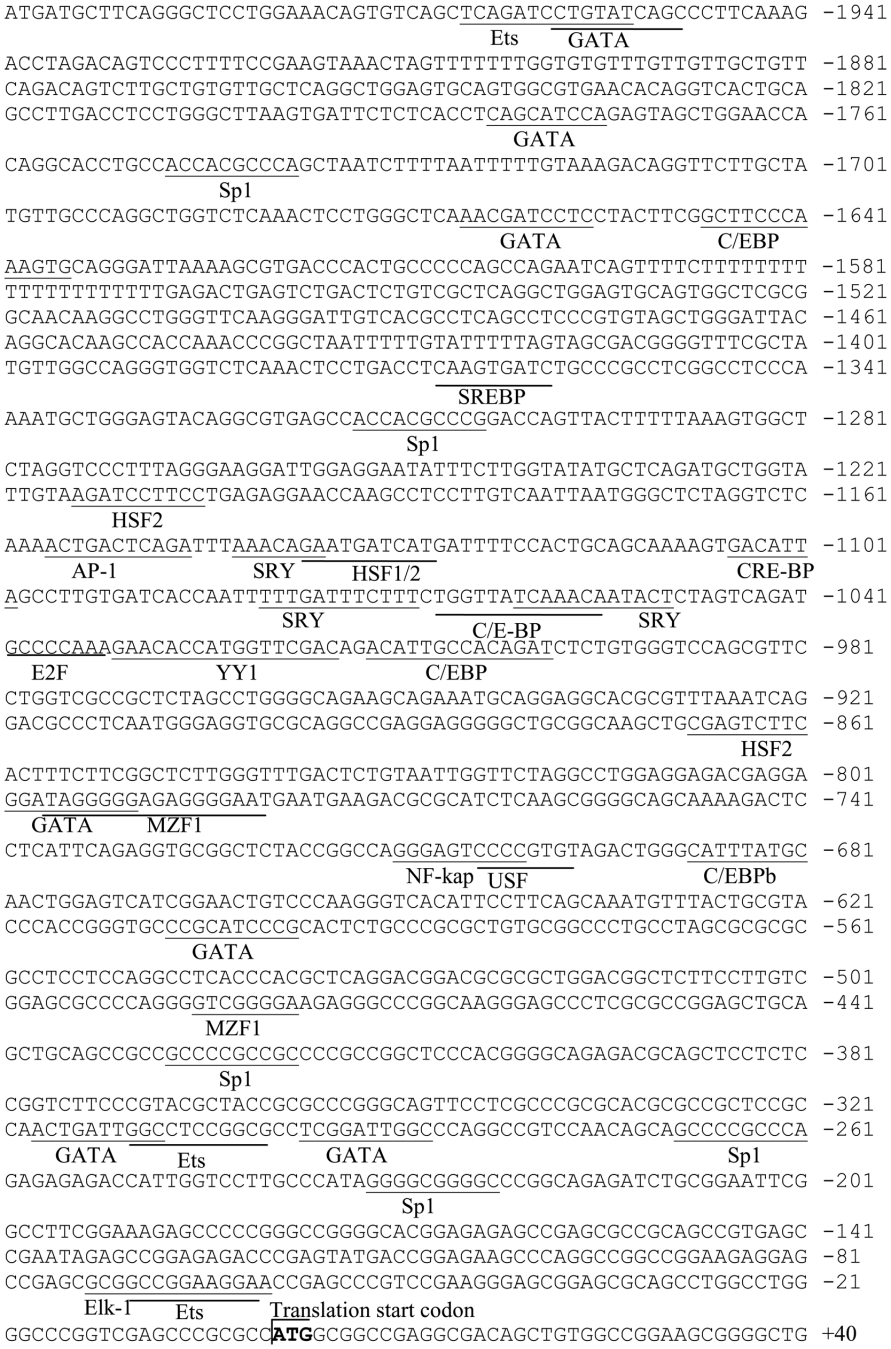
Sequence analysis of the *ckβ* 5′ flanking region. The predicted transcription factor binding sites are underlined. The translation start codon (ATG) is indicated in boldface.

**Figure 2:**
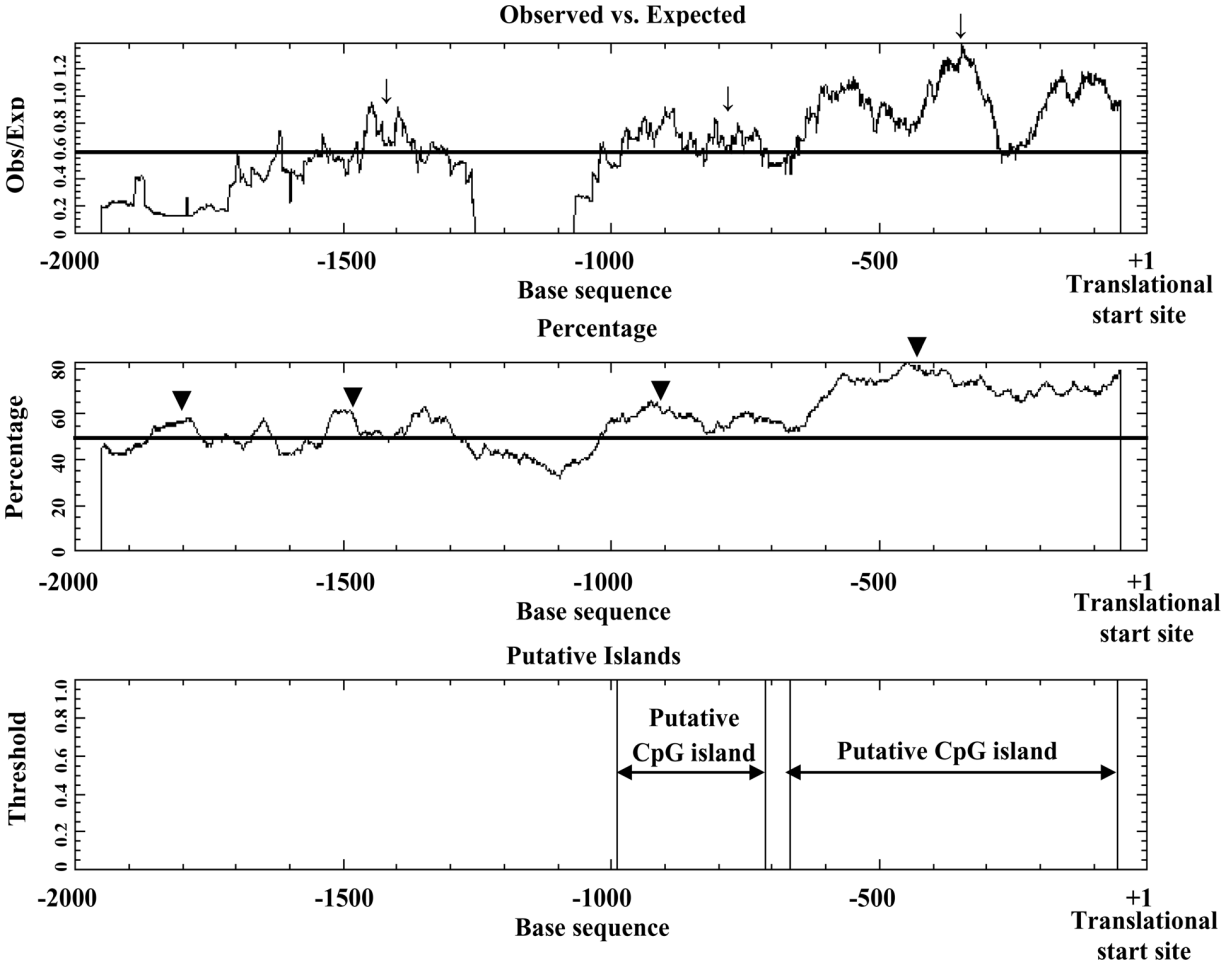
Identification of CpG island in the promoter region of *ckβ* gene by using CpGPlot. Regions with ≥200 bp of nucleotides, observed/expected ratio>0.6 (↓) and GC% >50% (▾) were identified as putative CpG islands.

A 2000 bp region 5′ of the human *ckβ* gene was first analyzed *in silico*. For convenient description hereafter, the nucleotide A at the ATG translational start site of the *ckβ* gene was designated +1, and nucleotides upstream of +1 were assigned negative numbers. The putative promoter sequence was analyzed for potential transcription factor binding sites using MatInspector 8.0 and TFSEARCH (Fig. 1). The promoter region contains several potential binding sites for Sp1, Ets, GATA factors, SREBP, MZF1, NF-κB, AP-1, and E2F (Fig. 1). The analysis showed that the *ckβ* promoter contains neither a CAAT box nor a recognizable consensus TATA box in close proximity to the transcription start site, which is typical of GC-rich promoters. The presence of numerous Sp1 binding sites indicates that the *ckβ* promoter contains a high GC content. Thus, CpGPlot and CpGIS were used to identify potential CpG island(s) in the *ckβ* promoter. Two potential CpG islands were identified by CpGPlot (Fig. 2), spanning the region between −989 and −713 (percent GC content: 59.21% and observed/expected ratio: 0.73) and the region between −666 and −56 (percent GC content: 71.52% and observed/expected ratio: 0.94). CpGIS analysis identified one potential CpG island covering both the putative CpG islands that were predicted by CpGPlot. This CpG island is 1085 bp long, stretches from −1085 to −1, and has a GC percentage of 65.6% and an observed/expected ratio of 0.864.

### Repressive regions are identified at the *ckβ* promoter

**Figure 3:**
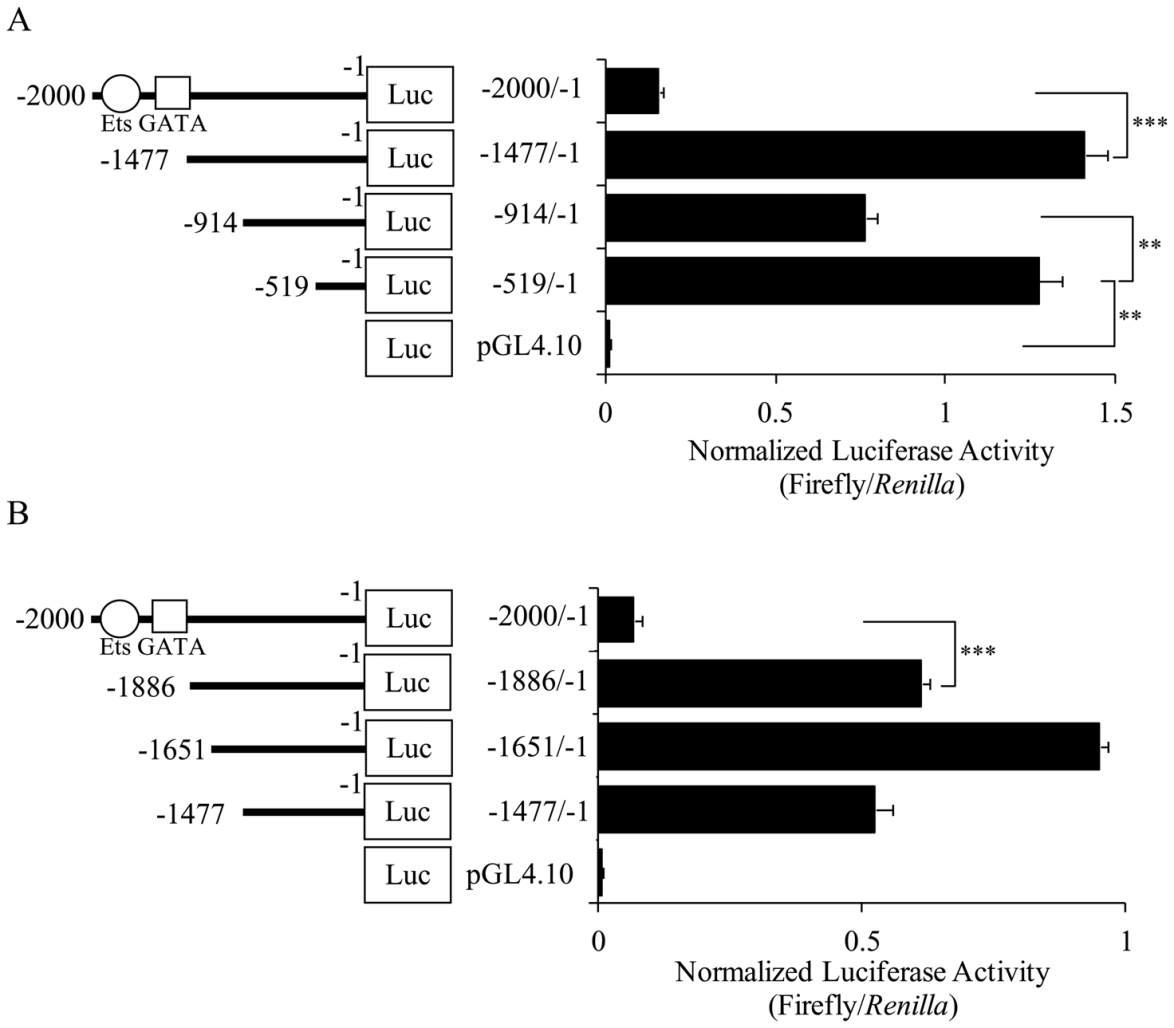
Deletion analysis of the *ckβ* promoter in MCF-7 cells. (A) Results from the first round of luciferase assays with 5′-truncated *ckβ* promoter constructs (ranging from −2000 to −519). (B) Results from the second round of luciferase assays with 5′-truncated *ckβ* promoter constructs (ranging from −2000 to −1477). The schematic structures of the step-wise 5′ deletion constructs are shown on the left. Ets and GATA binding sites investigated in this study are indicated by an open circle and open square, respectively. Each bar represents the mean ±SEM from three independent experiments performed in triplicate (*p<0.05; **p<0.01; and ***p<0.001).

To identify the important *cis*-acting regulatory elements in the *ckβ* promoter, various fragments of the *ckβ* promoter were cloned upstream of the firefly luciferase reporter vector. An initial deletion analysis of the *ckβ* promoter was performed with four *ckβ* promoter constructs [pGL4.10-*ckβ*(−2000/−1), pGL4.10-*ckβ*(−1477/−1), pGL4.10-*ckβ*(−914/−1), and pGL4.10-*ckβ*(−519/−1)] that contain promoter fragments of different lengths. Two repressive regions were identified in the first deletion analysis (Fig. 3A). Deletion of the 5′ sequence from the parental promoter construct to position −1477 resulted in a dramatic increase (809%) in promoter activity compared to that of the full-length 2000 bp promoter. Deletion of the region between −519 to −914 increased the promoter activity by 66%. This result showed that there is at least one inhibitory element located within the region of −2000/−1477 and another within −914/−519. The deletion analysis also showed that the basal promoter activity of *ckβ* was dependent on the promoter region between −519 and −1. Subsequently, another series of deletion mutants were constructed to narrow down the region between −2000 and −1477 that contained the inhibitory element(s). As shown in Fig. 3B, deletion of the sequence between −2000 and −1886 increased the promoter activity by 800%, demonstrating that this region significantly repressed the transcription of the *ckβ* promoter. Our subsequent experiments focused on the Ets (−1954/−1966) and GATA (−1950/−1959) binding elements located in this region, which are simply referred to as “Ets” and “GATA” in all subsequent parts of this study.

### Ets and GATA elements are responsible for the repression of *ckβ* promoter activity

**Figure 4:**
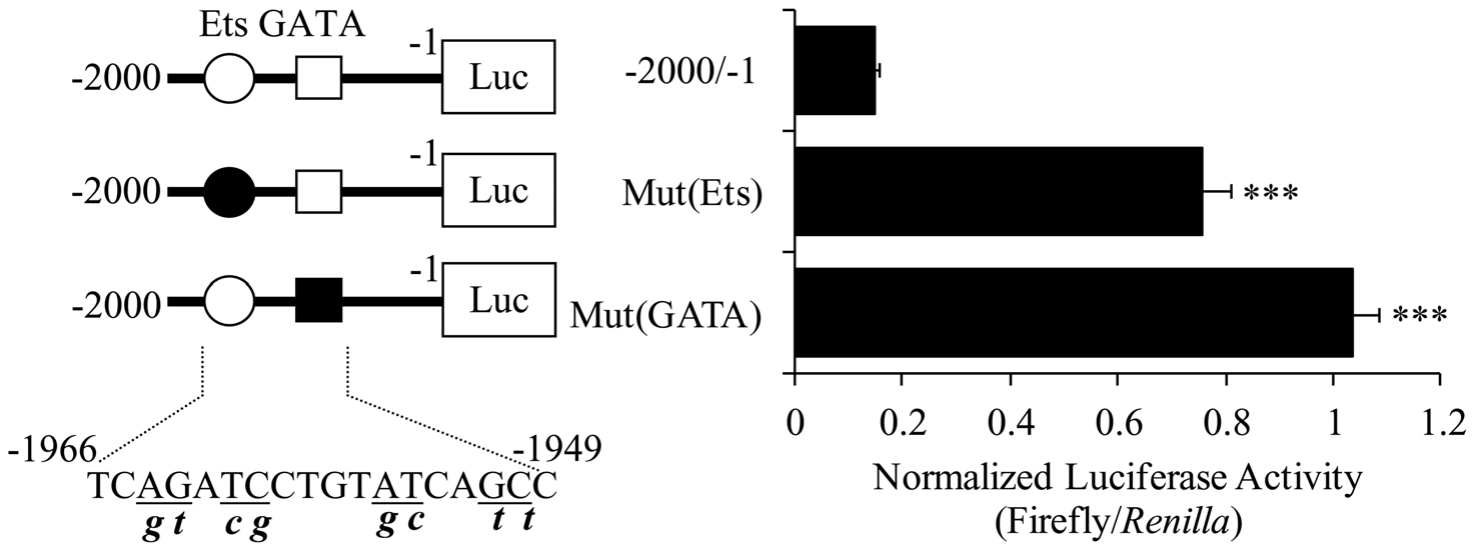
Effect of Ets and GATA binding site mutations on *ckβ* promoter activity. Ets and GATA binding sites are indicated with a circle and square, respectively. Mutated sites are indicated with closed circles and squares. The mutations introduced into the binding sites (underlined) are in lowercase. Each bar represents the mean ±SEM from three independent experiments performed in triplicate [***p<0.001 vs. pGL4.10-*ckβ*(−2000/−1)].

Bioinformatic analysis revealed that a canonical Ets binding site and a GATA transcription factor binding site were located within the −2000/−1886 region (Fig. 1). Therefore, point mutations were introduced in these binding sites to investigate the importance of specific sequences in the repression of the *ckβ* promoter. Mutation of the Ets element increased *ckβ* promoter activity to 513% of the wild-type promoter activity, while disruption of the core sequence of the GATA binding site markedly increased the promoter activity to 703% of the wild type (Fig. 4). Results from these experiments were consistent with the deletion analysis, in which the removal of both the Ets and GATA binding sites (5′ deletion from −2000 to −1886) caused a significant loss of *ckβ* promoter repression. Our data suggest that both the GATA and Ets sites located between −2000 and −1886 are negative regulatory elements and these two factors may interact to repress *ckβ* promoter activity.

### Ets and GATA3 transcription factors bind to the *ckβ* distal promoter

**Figure 5:**
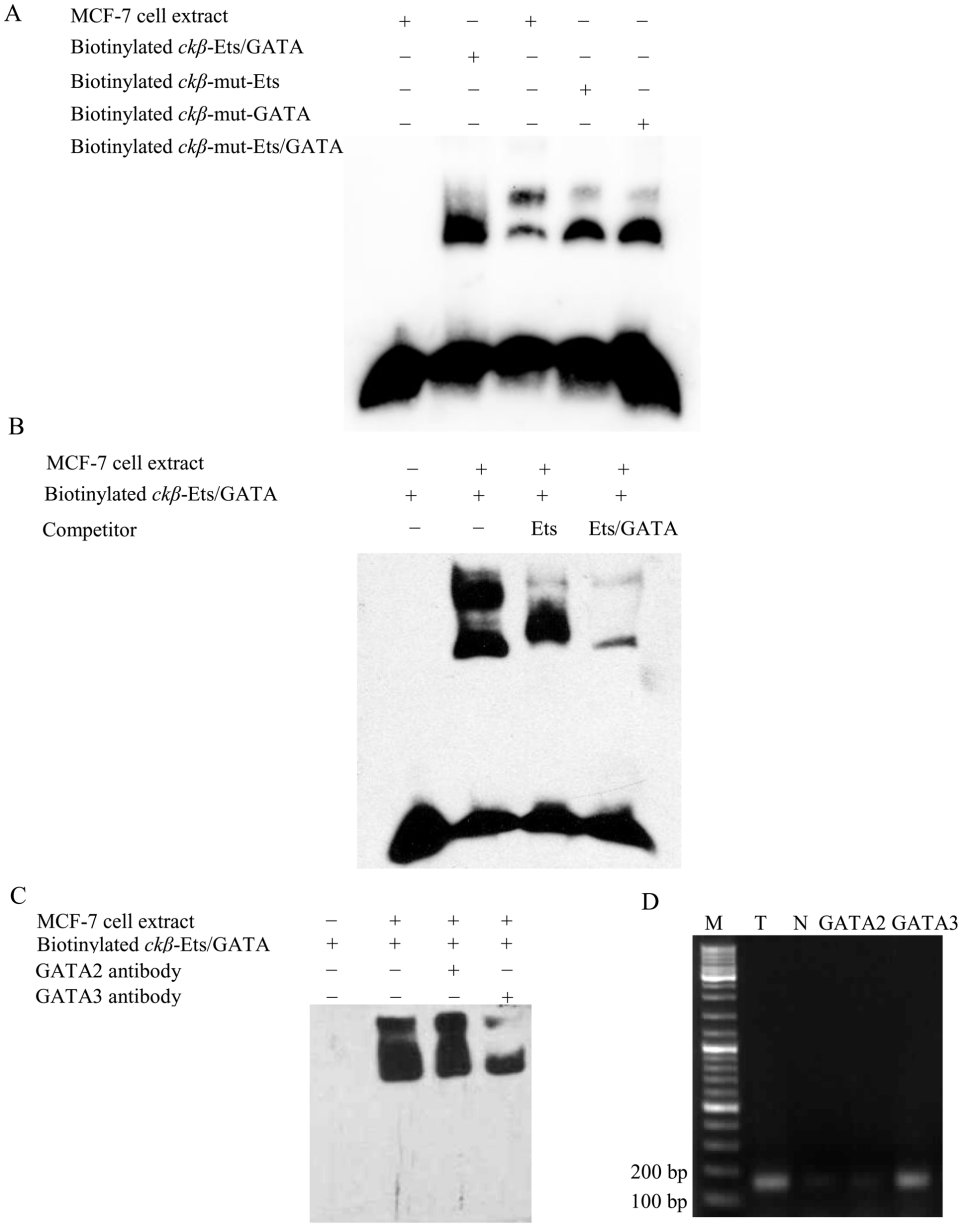
Characterization of Ets and GATA binding to the *ckβ* promoter by EMSA and ChIP. (A) EMSA was performed by using biotinylated *ckβ*-Ets/GATA probes with or without mutations at the Ets and GATA binding sequence. The blot shown is representative of two independent experiments that produced similar results. (B) Competition assay was performed by using unlabeled Ets and Ets/GATA consensus probes as competitors in the EMSA. The blot shown is representative of three independent experiments that produced similar results. (C) Supershift assay was performed by using GATA2 or GATA3 antibody in the EMSA. The blot shown is representative of two independent experiments that produced similar results. (D) ChIP was performed with GATA2 or GATA3 antibody and pre-immune normal rabbit IgG as control. Lane M: GeneRuler DNA Ladder Mix, Lane T: total input positive control (unprocessed chromatin), Lane N: pre-immune normal rabbit IgG (negative control).

EMSAs with a biotin-labeled DNA probe were used to assess the binding of the Ets and GATA repressive elements (between −2000 and −1886 in the *ckβ* promoter) by transcription factors from nuclear extracts from MCF-7 cells. Fig. 5A shows that the biotin-labeled DNA probe produced two slowly migrating shifted complexes, indicating two different nuclear proteins were bound to the probe. Mutations of either Ets, GATA or both elements in the promoter sequence markedly reduced the formation of the upper shifted complex, suggesting that the protein component of the complex was Ets- and GATA-related.

The binding specificity of Ets and GATA in the shifted complexes was verified by competition binding assays with DNA probes containing consensus binding sequences for Ets [25] and Ets/GATA [26]. The core motif, (A/T)GATA(A/G) in the Ets/GATA probe is recognized by six members (GATA1 to GATA6) of GATA-family transcription factors [27], [28]. The competition assay presented in Fig. 5B showed that the upper shifted complex was nearly eliminated by a 100-fold molar excess of unlabeled Ets consensus probe, while both shifted complexes were significantly reduced when the unlabeled Ets/GATA consensus probe was included in the binding reaction. These results demonstrated that the upper shifted complex was due to the Ets transcription factor and that both Ets and GATA transcription factors were bound to the repressive elements on the *ckβ* distal promoter.

Next, GATA2 and GATA3 antibodies were used in a supershift assay to determine if any of these two GATA transcription factors was involved in the formation of complex with the *ckβ* promoter. As shown in Fig. 5C, GATA2 antibody did not change the mobility and intensity of the DNA-protein complexes. The addition of GATA3 antibody into the EMSA did not produce other slower-migrating complex. However, it reduced the intensity of both the original upper and lower shifted complexes possibly by disrupting the interaction of GATA3 with *ckβ* promoter. The results showed that GATA3 but not GATA2 was bound to the *ckβ* distal promoter. Unbound probes were not observed in Fig. 5C because the gel was run for extended duration in order to increase the possibility of detecting other slower-migrating complexes. Similar results (reduced intensity of shifted complexes) were also obtained when the gel was not over-run and the free probes were still visible in an earlier supershift experiment with GATA3 antibody (S1 Figure).

Chromatin immunoprecipitation assay was performed to confirm the intracellular binding of GATA3 transcription factor to the *ckβ* distal promoter. Protein-DNA complexes were immunoprecipitated with GATA2 or GATA3 antibodies, followed by PCR amplification using the primers flanking the GATA binding site of the *ckβ* promoter. Fig. 5D showed that immunoprecipitate obtained with GATA3 antibody yielded a prominent PCR product at the expected size of 150 bp, whereas immunoprecipitates obtained with pre-immune rabbit IgG and GATA2 antibody produced much lower amount of amplified products. Sequencing of the PCR product obtained with GATA3 immunoprecipitate confirmed the specificity of the reaction. These results further support the binding of GATA3 to the GATA binding site in *ckβ* distal promoter.

### PMA represses *ckβ* promoter activity

**Figure 6:**
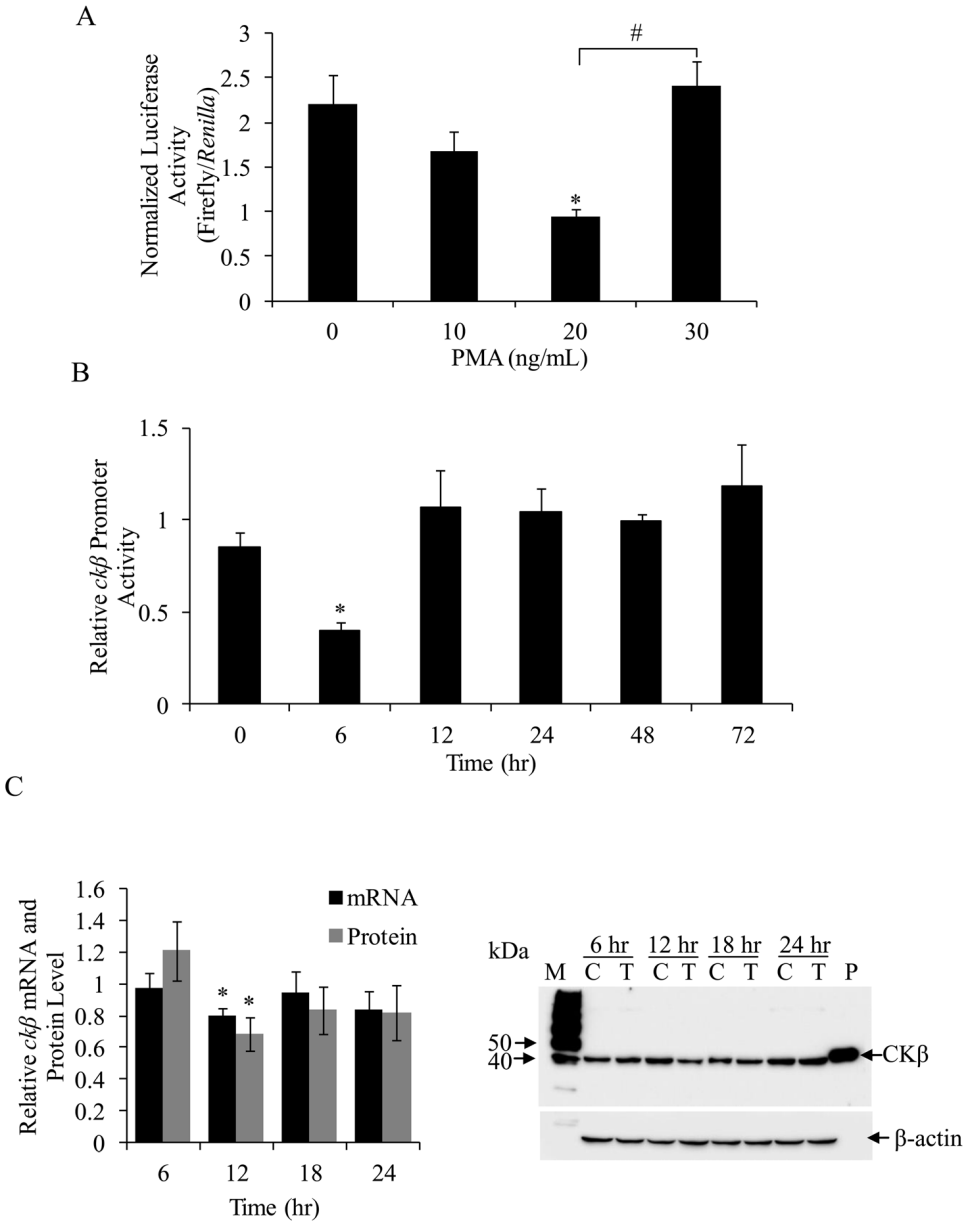
Effects of PMA concentration and treatment duration on *ckβ* promoter activity. (A) MCF-7 cells were treated with 10, 20, or 30 ng/mL PMA for 6 hr. Each bar represents the mean ±SEM of triplicate samples from three independent experiments. (*p<0.05 vs. DMSO control; #*p*<0.05, within the PMA treatment group). (B) MCF-7 cells were treated with 20 ng/mL of PMA for 6 to 72 hr. The values shown are *ckβ* promoter activities relative to their respective controls (control value is set at 1.0, *p<0.05 vs. DMSO control). Each bar represents the mean ±SEM of triplicate samples from three independent experiments. (C) Effect of PMA treatment on the *ckβ* mRNA and protein levels in MCF-7 cells. Cells were treated with 20 ng/mL of PMA or DMSO for 6 to 24 hr. Left panel: The levels of mRNA and proteins are relative to their respective controls (control value is set at 1.0, *p<0.05 vs. DMSO control). Each bar represents the mean ±SEM of triplicate samples from three independent experiments. Right panel: The blot shown is representative of three independent experiments that produced similar results. M: SuperSignal Molecular Weight protein ladders (Thermo Scientific, USA), C: control, T: PMA treated, P: purified CKβ protein (45 kDa) as reference.

PMA regulates certain Ets and GATA family transcription factors by activating the PKC-mediated mitogen-activated protein kinase (MAPK) pathway [29], [30]. This prompted us to examine the effect of PMA on *ckβ* promoter activity. The effects of PMA on PKCs are dependent on the PMA concentration and the duration of treatment. Short-term treatment with a low concentration of PMA activates PKCs, while long-term exposure to a high concentration has an inhibitory effect on PKCs [31], [32]. In this study, MCF-7 cells were treated with different concentrations of PMA (10, 20, and 30 ng/mL) for 6 hr after transfection with the wild-type pGL4.10-*ckβ*(−2000/−1) reporter plasmid. PMA repressed *ckβ* promoter activity in MCF-7 cells starting at 10 ng/mL and reaching a maximal effect at 20 ng/mL (Fig. 6A). However, a 6 hr exposure to 30 ng/mL PMA attenuated the repression of *ckβ* promoter activity. The effect of PMA treatment duration on *ckβ* promoter activity was examined in the next series of experiments, ranging from 6 to 72 hr with a PMA concentration of 20 ng/mL. As shown in Fig. 6B, the maximum repressive effect of PMA occurred at 6 hr after treatment (approximately 38% of the DMSO control). Promoter activity began to increase and was almost equal to that of the DMSO control treatment after 12 hr of PMA treatment. Overall, chronic or extended PMA treatment eliminated the PMA effect on the *ckβ* promoter activity, suggesting that PMA-sensitive PKC isozymes are involved in the PMA-mediated repression of the *ckβ* promoter. This result also ruled out the involvement of PKCζ, as this isozyme is known to be resistant to chronic PMA treatment [31], [33].

The effect of PMA treatment on endogenous *ckβ* gene expression was also investigated. Real-time PCR quantifications of *ckβ* mRNA and Western detection of CKβ protein were performed on DMSO (control) and PMA treated (20 ng/mL for 6 to 24 hr) MCF-7 cells. Based on the results shown in Fig. 6C, PMA treatment resulted in significant reduction of *ckβ* mRNA (by 20%) and protein (by 30%) expressions compared to controls after 12 hr. There was no significant difference for mRNA and protein levels between PMA treated samples and controls for the other treatment durations.

### PKC412 and PKCε inhibitor peptide abolished the repressive effect of PMA on *ckβ* promoter activity

**Figure 7:**
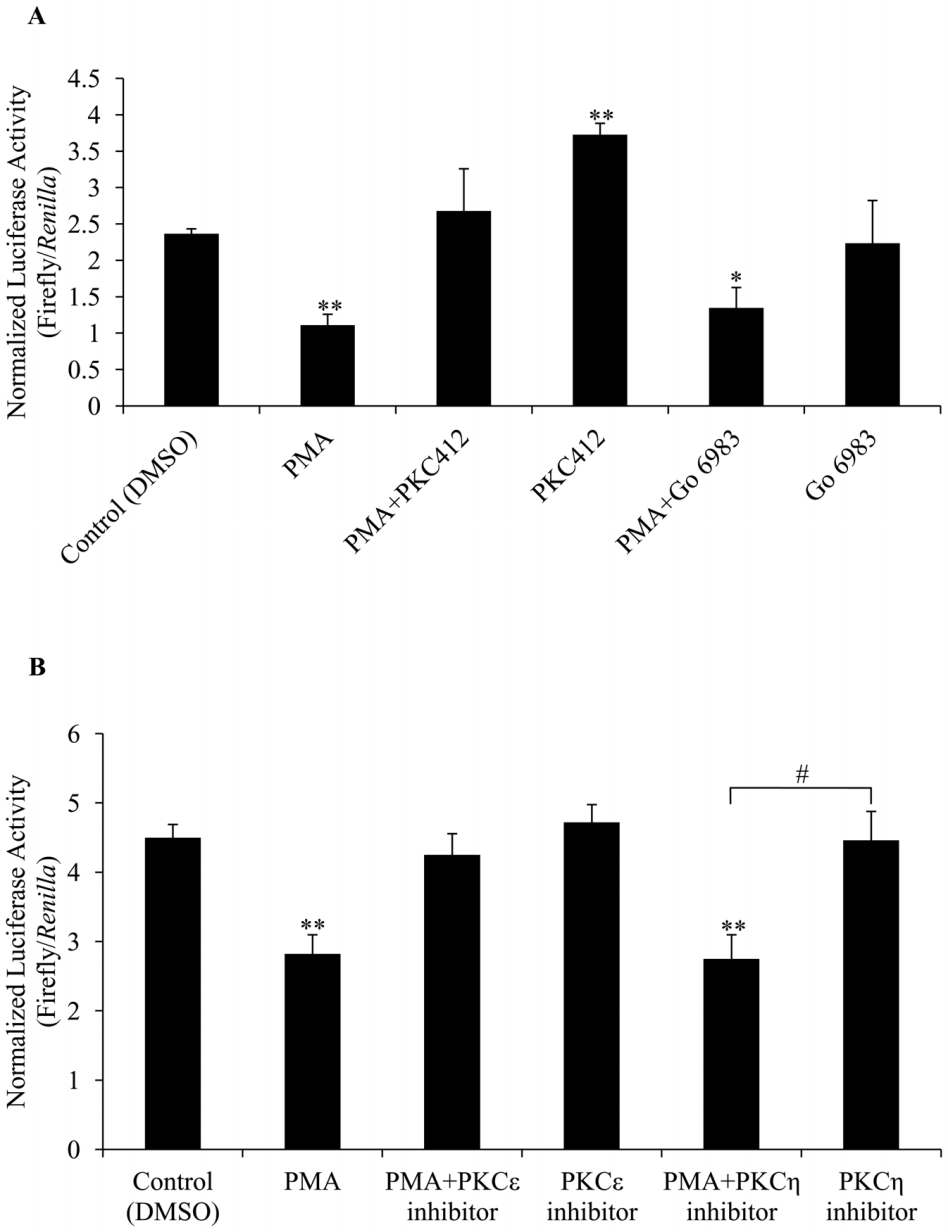
Effect of PKC inhibitors (PKC412 and Go 6983) on the PMA-mediated repression of *ckβ* promoter activity. (A) MCF-7 cells were individually or co-treated with 20 ng/mL PMA, 1 mM PKC412 and 0.1 mM Go 6983 for 6 hr. Each bar represents the mean ±SEM of triplicate samples from three independent experiments. (*p<0.05 and **p<0.01 vs. DMSO control). (B) MCF-7 cells were individually or co-treated with 20 ng/mL PMA, 10 µM PKCε inhibitor peptide and 10 µM PKCη pseudo-substrate inhibitor for 6 hr. Each bar represents the mean ±SEM of triplicate samples from three independent experiments. (*p<0.05 and **p<0.01 vs. DMSO control, #*p*<0.05 between the two indicated treatments).

PKC412 (midostaurin, or CGP 41251), an inhibitor of PKCα, −β, −γ, −δ, −ε, −η, and −ζ [34], completely abolished the PMA-induced down-regulation of the *ckβ* promoter, whereas Go 6983, an inhibitor of PKCα, −β, −γ, −δ, and −ζ [32], [35], did not show the same effect (Fig. 7A). The *ckβ* promoter activity was also increased by the individual treatment with PKC412 but not Go 6983. These findings, along with the resistance of PKCζ to chronic PMA treatment, suggest that PKCε or PKCη is most likely the PKC isozyme that mediates the repressive effect of PMA in this system. Subsequently, PKCε and PKCη specific inhibitors were used to identify the isozyme involved. The results in Fig. 7B show that PKCε inhibition abolished the PMA repression of *ckβ* promoter. PKCη inhibition did not affect the down-regulation of *ckβ* promoter by PMA treatment. The promoter activity of treatment with PMA and PKCη inhibitor was also significantly lower than treatment with PKCη alone. This further excludes the involvement of PKCη isozyme in the PMA repression of *ckβ* promoter.

### Ets and GATA binding is required for PMA-mediated repression of the *ckβ* promoter

**Figure 8:**
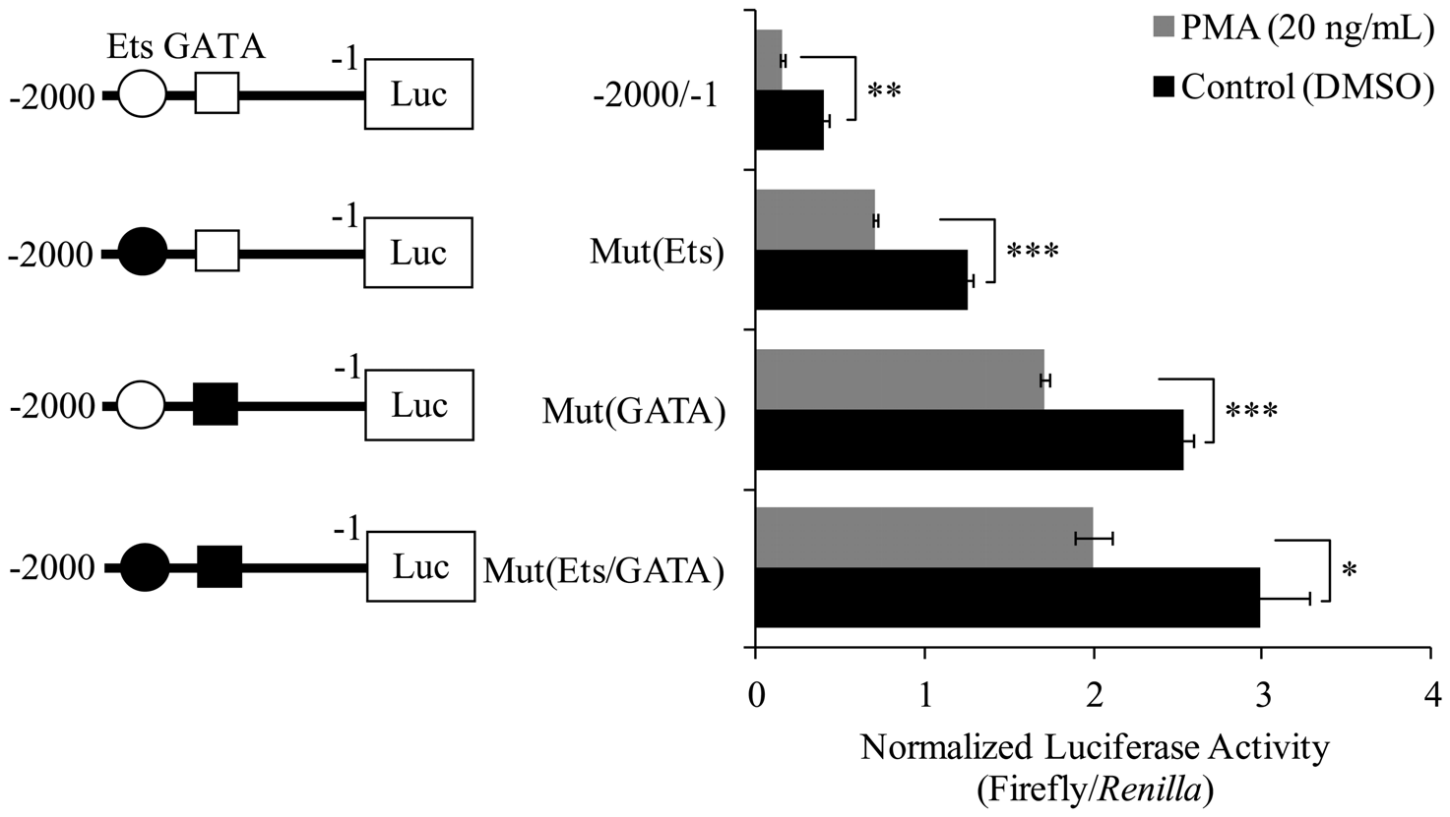
Effect of PMA on the activity of wild-type, Ets-mutated, GATA-mutated, and Ets/GATA mutated *ckβ* promoter constructs. Each bar represents the mean ±SEM of triplicate samples from three independent experiments. (*p<0.05, **p<0.01, and ***p<0.001 vs. DMSO control).

To investigate the role of the Ets and GATA binding sites in the PMA-mediated repression of the *ckβ* promoter, the wild-type promoter, pGL4.10-*ckβ*(−2000/−1), and constructs in which the Ets and GATA binding sites were mutated were transfected into MCF-7 cells followed by treatment with or without PMA. Fig. 8 shows that PMA treatment decreased the promoter activities of pGL4.10-*ckβ*(−2000/−1), pGL4.10-mut(Ets), and pGL4.10-mut(GATA) to 38%, 57%, and 68%, respectively, as compared to the control (DMSO treatment). This result indicated the participation of both Ets and GATA in the *ckβ* promoter repression by PMA. The involvement of other transcription factors in PMA-mediated downregulation of *ckβ* promoter activity cannot be ruled out because the mutation of both the Ets and GATA sites did not completely abolish the effect of PMA treatment (Fig. 8).

**Figure 9:**
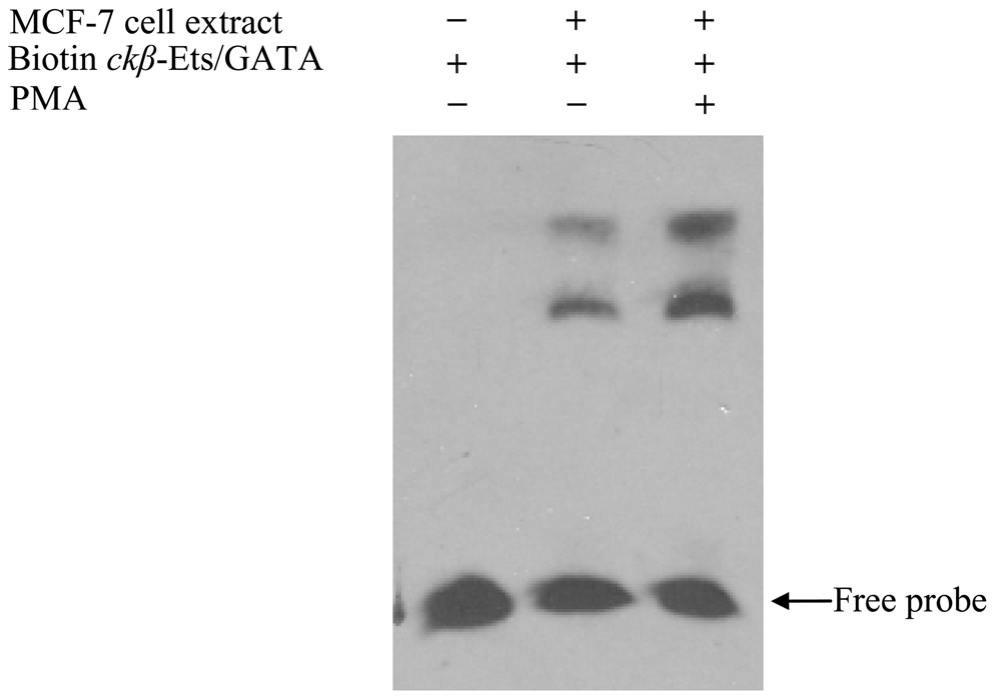
Effect of PMA on the binding of Ets and GATA to the *ckβ* distal promoter. EMSA was performed using nuclear extracts from MCF-7 cells treated with or without 20 ng/mL PMA. The blot is representative of three independent experiments that produced similar results.

EMSA was performed to investigate the effect of PMA treatment on Ets and GATA binding to the *ckβ* promoter. As shown in Fig. 9, PMA increased the binding of Ets and GATA transcription factors to the *ckβ* promoter and thereby suppressed its activity. Current data provide evidence that supports the Ets and GATA binding proteins as the key transcription factors involved in the PMA-mediated down-regulation of the *ckβ* promoter via activation of the PKC signaling pathway.

## Discussion

In this study, the promoter region of the *ckβ* gene was isolated and characterized to understand the transcriptional regulation of this enzyme. We have previously shown that a promoter region located 2000 bp upstream of the *ckβ* start codon was transcriptionally active in MCF-7 cells [17]. The *ckβ* promoter is a TATA-less, GC-rich promoter that may belong to the family of housekeeping gene promoters. In general, promoters that lack TATA-binding sites but contain CpG islands contain multiple GC box motifs that serve as binding sites for the Sp1 transcription factor [36], [37]. The ubiquitous transcription factor Sp1 serves as a constitutive activator of housekeeping genes by recruiting TATA-binding protein (TBP) to promoters lacking a recognizable consensus TATA box [37]. These observations are in agreement with our preliminary sequence analysis showing that the *ckβ* promoter contains multiple Sp1 binding sites, which reflects the GC-rich nature of this region.

In this study, Ets and GATA binding sites located in the *ckβ* distal promoter region were shown to be essential for the repression of this promoter's activity. Several Ets-related and GATA-related transcription factors are downstream transcriptional effectors in the PKC-dependent pathway, which is inducible by PMA [29], [30]. PMA stimulates PKC by binding to the C1 region of the PKC regulatory domain [38]. This knowledge prompted us to analyze the transcriptional regulation of the *ckβ* gene by PMA. Two observations supported the involvement of PKC in the PMA-mediated repression of *ckβ* promoter activity: (i) sensitivity to chronic PMA treatment and (ii) inhibition of the PMA-mediated effect by the PKC inhibitor PKC412. PKC is a serine/threonine-specific protein kinase comprising at least 12 related isozymes that can be categorized into three groups [39]. The “conventional” PKC members (cPKCs α, ΒI, ΒII, and γ) are activated by Ca^2+^, phosphatidylserine, and diacylglycerol/phorbol esters. Members of the second group, known as “novel” PKCs (nPKCs δ, ε, η, and θ), are unresponsive to Ca^2+^ but are stimulated by phosphatidylserine and diacylglycerol/phorbol esters. The third group, “atypical” PKCs (aPKCs λ and ζ), are activated by phosphatidylserine but are unresponsive to both Ca^2+^ and diacylglycerol/phorbol esters. PKCµ is a member of the PKC family that does not fit into any of the known PKC subgroups [40]. In the current study, it was deemed important to identify the main PKC isozyme involved in the PMA-mediated repression of the *ckβ* promoter to better elucidate the signaling mechanism that regulates *ckβ* transcription. The PKCθ isozyme is not expressed in MCF-7 cells [41]. Therefore, the involvement of PKCθ in the transcriptional regulation of *ckβ* can be ruled out because MCF-7 cells were used in this work. All PKC isozymes are sensitive to chronic PMA treatment except PKCζ [31], [33]. We observed that chronic PMA treatment led to complete abrogation of the PMA-mediated repression of the *ckβ* promoter, demonstrating that PMA-sensitive PKC(s) participated in the negative regulation of the *ckβ* promoter and ruled out the involvement of the PKCζ isozyme (because of its PMA resistance). The PKC isozymes responsible for the suppression of the *ckβ* promoter can be narrowed down to either PKCε or PKCη based on the complete abolishment of the PMA-mediated effect after treatment with PKC412 but not Go 6983, which does not target these two isozymes. Our subsequent experiments using specific inhibitors for the two isozymes revealed that PKCε was the PKC isozyme involved in the PMA-induced suppression of *ckβ* promoter. Previously, Kobayashi *et al*. [42] reported that PMA treatment of bipolar undifferentiated CG-4 cell line down regulated PKCα and PKCβ_II_ more rapidly than PKCε, which was still detectable by Western blot after 12 hr PMA treatment. On the contrary, the results in this study (Fig. 6B) showed that the PMA-mediated repression of *ckβ* promoter activity was abolished after 12 hr PMA treatment, indicating that the PKCε could be down regulated more rapidly by PMA treatment in MCF-7 cells than in the cell line used by Kobayashi *et al*. The effects of PMA treatment on *ckβ* mRNA and protein levels were slower (12 hr) and less pronounced than its effect on promoter activity (6 hr). However, both followed the same trend whereby chronic PMA treatment abolished the effects. Since the samples were taken every six hours, lower *ckβ* mRNA and protein levels caused by the strong suppression of promoter activity might be detected between 6 to 12 hr PMA treatments.

The present study shows that *ckβ* promoter activity was regulated by a PMA-induced PKC-dependent pathway. Mutation of Ets and GATA binding elements located on region −1950 to −1966 of the *ckβ* promoter confirmed the importance of these two binding sites for the PMA-induced, PKC-dependent repression of the *ckβ* promoter. The importance of these Ets and GATA elements was further demonstrated by EMSA results showing that PMA treatment increased the binding of both Ets and GATA factors to their respective binding elements on the *ckβ* promoter. It must be noted that a minor participation of other elements at the same promoter region cannot be excluded, as mutation of the Ets/GATA elements did not completely abolish the effect of PMA. Supershift assay was not performed to identify the binding protein of Ets motif in *ckβ* promoter from more than 30 Ets transcription factors in human [43]. However, we predicted that Ets1 and Ets2 are the potential binding proteins of the Ets site based on their overexpression in breast carcinomas like MCF-7 used in this study [44], [45] and their regulation by PMA through PKC pathway activation [30], [46]. Although this study clearly indicated that Ets and GATA transcription factors are required for PMA-induced repression of the *ckβ* promoter, the underlying mechanism of PKC involvement remains to be established. GATA family transcription factors are regulated by PMA via the MAPK signaling pathway [29], [47]. PMA has been shown to directly induce GATA4 phosphorylation via activation of the MAPK family member ERK, without involvement of the PKC signaling pathway [29]. By contrast, Li *et al*. [47] showed that PMA repressed erythroid differentiation-associated gene (EDAG) expression via the PKC-MAPK-GATA1 pathway. The abolishment of the PMA effect on the *ckβ* promoter by the PKC inhibitors PKC412 and PKCε inhibitor peptide suggested that PMA-induced binding of GATA transcription factors was mediated by MAPKs through a PKCε-dependent signaling pathway. Similarly, PMA activated PKCε to stimulate endothelin-converting enzyme 1 (ECE-1) activity through an MAPK-dependent Ets-1 pathway [48]. Activation of PKCε by PMA also induced translocator protein (*TSPO*) gene expression through the increased binding of c-jun and Ets-related GA-binding protein (GABP) to the AP-1 and Ets sites, respectively, on the *TSPO* promoter [49], [50]. The MAPK (Raf-1-MEK1/2-ERK1/2) pathway was identified as the downstream target of PKCε that mediates the PMA effect on *TSPO* gene expression [50]. These observations support the hypothesis in this study that the binding of Ets and GATA transcription factors to the *ckβ* promoter is induced by PMA through the activation of PKCε and MAPKs.

The activity of Ets family transcription factors is regulated by their interactions with other adjacently located transcription factors [51]. The formation of protein-protein complexes on the promoter can modulate the transcriptional activation or repression properties of the two partners and allow for crosstalk between different signal transduction pathways [43]. Functional interactions between Ets1/Ets2 and GATA3 have been shown to synergistically increase the activity of the human interleukin-5 promoter in the presence of PMA [52]. Likewise, in this study Ets- and GATA-related proteins could synergistically and cooperatively repress *ckβ* promoter activity due to their adjacently located binding sites.

In summary, this is the first report linking transcriptional regulation of *ckβ* to the PKC signaling pathway. We postulate that PMA-induced Ets and GATA transcription factors binding to the region between −1950 and −1966 of the *ckβ* promoter represses its activity via the activation of PKCε in MCF-7 cells.

## Supporting Information

S1 Figure **Characterization of GATA binding to the** ***ckβ*** **promoter by supershift assay.** Supershift assay was performed by using 2 or 5 µg of GATA3 antibody in the EMSA. The electrophoresis was stopped when the bromophenol blue dye has reached 3/4 of the length of the gel. The blot shown is representative of two independent experiments that produced similar results.
(TIF)
