# Supplementary figures and images for: Ets and GATA Transcription Factors Play a Critical Role in PMA-Mediated Repression of the ckβ Promoter via the Protein Kinase C Signaling Pathway
Source: PLoS One. 2014 Dec 9;9(12):e113485. doi: 10.1371/journal.pone.0113485 (PMC4260826; doi:10.1371/journal.pone.0113485)

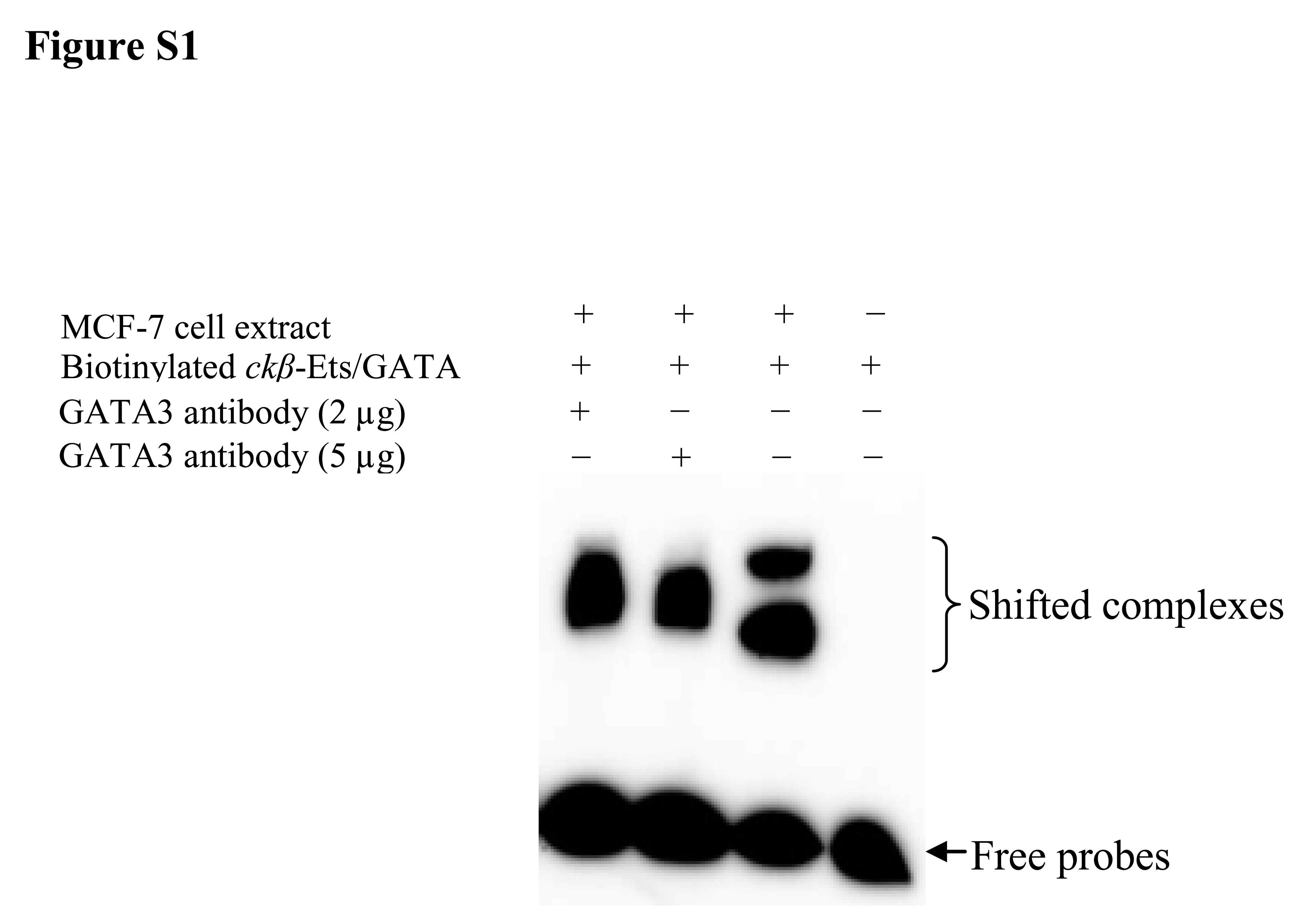

Supplement: S1 Figure — Characterization of GATA binding to the ckβ promoter by supershift assay. Supershift assay was performed by using 2 or 5 µg of GATA3 antibody in the EMSA. The electrophoresis was stopped when the bromophenol blue dye has reached 3/4 of the length of the gel. The blot shown is representative of two independent experiments that produced similar results. (TIF) [file pone.0113485.s001.tif]
